# Block Copolymer Micelles Encapsulating Au(III) Bis(Dithiolene) Complexes as Promising Nanostructures with Antiplasmodial Activity

**DOI:** 10.3390/pharmaceutics15031030

**Published:** 2023-03-22

**Authors:** Joana F. Santos, Raquel Azevedo, Miguel Prudêncio, Fernanda Marques, Yann Le Gal, Dominique Lorcy, Célia Fernandes

**Affiliations:** 1 Centro de Ciências e Tecnologias Nucleares, Instituto Superior Técnico, Universidade de Lisboa, Estrada Nacional 10, km 139.7, 2695-066 Bobadela, Portugal; joana.f.santos@tecnico.ulisboa.pt (J.F.S.); fmarujo@ctn.tecnico.ulisboa.pt (F.M.); 2 Instituto de Medicina Molecular João Lobo Antunes, Faculdade de Medicina, Universidade de Lisboa, Av. Prof. Egas Moniz, 1649-028 Lisboa, Portugal; raquel.azevedo@medicina.ulisboa.pt (R.A.); mprudencio@medicina.ulisboa.pt (M.P.); 3 Departamento de Engenharia e Ciências Nucleares, Instituto Superior Técnico, Universidade de Lisboa, Estrada Nacional 10, km 139.7, 2695-066 Bobadela, Portugal; 4 Institut des Sciences Chimiques de Rennes-UMR 6226, Université de Rennes, CNRS, ISCR, F-35000 Rennes, France; yann.le-gal@univ-rennes1.fr (Y.L.G.); dominique.lorcy@univ-rennes1.fr (D.L.)

**Keywords:** block copolymer micelles, gold(III) bis(dithiolene) complexes, drug delivery, antimalarial drugs, antiplasmodial activity

## Abstract

Block copolymer micelles (BCMs) can be used to improve the solubility of lipophilic drugs and increase their circulation half-life. Hence, BCMs assembled from MePEG-*b*-PCL were evaluated as drug delivery systems of gold(III) bis(dithiolene) complexes (herein AuS and AuSe) to be employed as antiplasmodial drugs. These complexes exhibited remarkable antiplasmodial activity against liver stages of the *Plasmodium berghei* parasite, and low toxicity in a model of zebrafish embryos. To improve the complexes’ solubility, BCMs were loaded with AuS, AuSe, and the reference drug primaquine (PQ). PQ-BCMs (D_h_ = 50.9 ± 2.8 nm), AuSe-BCMs (D_h_ = 87.1 ± 9.7 nm), and AuS-BCMs (D_h_ = 72.8 ± 3.1 nm) were obtained with a loading efficiency of 82.5%, 55.5%, and 77.4%, respectively. HPLC analysis and UV–Vis spectrophotometry showed that the compounds did not suffer degradation after encapsulation in BCMs. In vitro release studies suggest that AuS/AuSe-BCMs present a more controlled release compared with PQ-loaded BCMs. The antiplasmodial hepatic activity of the drugs was assessed in vitro and results indicate that both complexes present higher inhibitory activity than PQ, although encapsulated AuS and AuSe presented lower activity than their non-encapsulated counterparts. Nevertheless, these results suggest that the use of BCMs as delivery vehicles for lipophilic metallodrugs, particularly AuS and AuSe, could enable the controlled release of complexes and improve their biocompatibility, constituting a promising alternative to conventional antimalarial treatments.

## 1. Introduction

Despite intense efforts toward the prevention of malaria, more than 200 million people are still affected by this disease annually [1]. Malaria is caused by *Plasmodium* spp. parasites, transmitted through the bite of female *Anopheles* mosquitoes [2]. Although chloroquine has been used for many years as the primary treatment for this disease, resistance to this drug has now spread in the most affected countries [3]. Primaquine (PQ) is also administered for malaria treatment but its haemolytic toxicity in people with glucose-6-phosphate dehydrogenase (G6PD) deficiency, a common genetic trait in malaria-endemic regions, limits its use [4]. Artemisinin is currently used for the treatment of malarial infections but recent studies suggest an emergence of partial resistance to this drug, highlighting the need to identify new effective drugs [5,6,7].

Gold(I/III) complexes have been shown to be promising candidates as antimalarial drugs. Auranofin, a gold(I) complex approved for the treatment of rheumatoid arthritis, displayed significant antiplasmodial effects, strongly inhibiting *P. falciparum* growth at low concentrations [8]; however, the commonly reported side effects such as skin lesions, oral ulcers, renal damage, and thrombocytopenia, among others, limits its application [9].

Several other gold(I/III) complexes bearing phosphine, N-heterocyclic carbenes, and thiosemicarbazonato ligands have also presented significant in vitro antiplasmodial activity comparable to that of clinically established antimalarial drugs [10,11,12,13]. Several studies indicate that gold compounds inhibit *P. falciparum* thioredoxin reductase, causing oxidative stress, which is thought to be responsible for the antiplasmodial effects observed [8,12]. However, further studies are needed to better understand the mechanism of action of these compounds.

Recently, the in vitro activity of several Au(III) bis(dithiolene) complexes against the hepatic stage of the rodent *P. berghei* infection has been evaluated, with the most promising compounds being also assessed against the blood stage of *P. falciparum* infection [14]. These complexes exhibited remarkable antiplasmodial activity against the liver stages of *Plasmodium berghei* parasites, a high antitumor effect on cancer cells, and very low toxicity in a model of zebrafish embryos, important properties for potential therapeutic applications.

**Figure 1:**
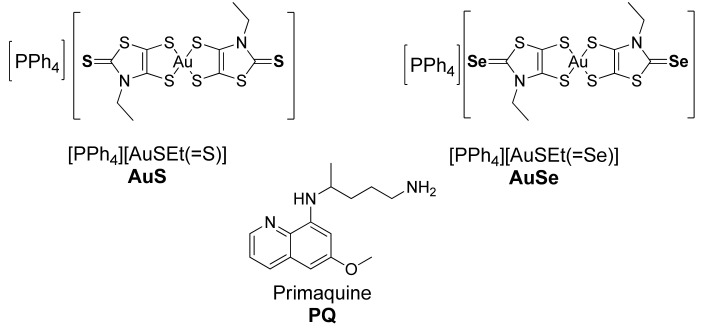
Chemical structures of the gold complexes, AuS and AuSe, and the reference drug PQ.

The compounds that presented the strongest antiplasmodial activity were the Au(III) bisdithiolene complexes, namely [PPh_4_][AuSEt(=Se)] and [PPh_4_][AuSEt(=S)], with IC_50_ values of 0.4 μM and 0.5 μM, respectively, against the hepatic stage of *P. berghei* infection, and 1 μM against the blood stage of *P. falciparum* infection (Figure 1) [14].

The main structural difference between these two complexes is the nature of the exocyclic chalcogen atom, which is a selenium one for [PPh_4_][AuSEt(=Se)] and a sulfur one for [PPh_4_][AuSEt(=S)]. However, the poor water solubility of these complexes, probably related to their planar molecular geometries, constrains their bioavailability, which represents a limitation to their efficacy in vivo. To overcome this issue, drug delivery systems such as block copolymer micelles (BCMs) can be used to increase the solubility of hydrophobic drugs. BCMs are constituted by amphiphilic copolymers with a hydrophobic core surrounded by a hydrophilic corona and self-assemble in an aqueous medium. Due to the structural arrangement of BCMs, it is possible to encapsulate hydrophobic drugs in their core, increasing drug solubility and, consequently, their half-life circulation in the blood [15,16].

There are few studies regarding the encapsulation of antimalarial drugs in BCMs. Artemisinin-loaded BCMs synthesized from diblock [17] and triblock [18] copolymers have shown a sustained in vitro release of artemisinin and a high loading efficiency. Moreover, in vivo studies showed that the parasite’s growth is more effectively inhibited by the loaded-triblock copolymer micelles than the free artemisinin [18]. Plumbagin was also encapsulated in PCL-PEG-PCL micelles with high loading efficiency, with the loaded micelles presenting a sustained drug release profile and in vivo studies suggesting an increased circulation half-life than that of the free drug [19].

Herein, we report the preparation and characterization of BCMs loaded with [PPh_4_][AuSEt(=Se)] (AuSe) and [PPh_4_][AuSEt(=S)] (AuS) using the copolymer MePEG-*b*-PCL (methoxy poly(ethylene glycol)-b-poly(ε-caprolactone). BCMs loaded with the standard antiplasmodial drug PQ were also prepared to be included as a positive control (Figure 1). The in vitro release of the drugs was evaluated in physiological conditions.

To determine the inhibitory activity of BCMs loaded with Au(III) bis(dithiolene) complexes on the hepatic stage of *Plasmodium*, AuSe and AuS were screened in parallel, with and without BCMs encapsulation, using an in vitro model of hepatic infection by *P. berghei* sporozoites and a human hepatoma cell line (Huh7).

## 2. Materials and Methods

### 2.1. Materials

All chemicals and solvents were of reagent grade and used without additional purification unless otherwise stated. Dichloromethane was acquired from Sigma-Aldrich (St. Louis, MI, USA) and dried using phosphorus pentoxide. Toluene was dried by distillation with sodium and was obtained from Fischer Chemical (Waltham, MA, USA). Poly(ethylene glycol) methyl ether (MePEG, Mn = 5000), ε-caprolactone (CL), and a 2 M solution of hydrogen chloride in diethyl ether were acquired from Sigma-Aldrich (St. Louis, MI, USA). CL was dried with calcium hydride and distilled prior to use. Me-PEG (Mn = 5000) was dried twice by azeotropic distillation in toluene that was distilled off completely. Dimethylformamide (DMF) and acetonitrile (ACN) were acquired from Fisher Chemical (Waltham, MA, USA) and Honeywell (Charlotte, NC, USA), respectively.

Primaquine diphosphate 98% was obtained from Sigma-Aldrich (St. Louis, MI, USA). The gold complexes AuSe and AuS were synthesized and characterized as previously described [14].

### 2.2. Methods

UV–Vis spectrophotometry was performed on a Cary 60 UV–Vis spectrophotometer from Agilent Technologies (Santa Clara, CA, USA) with quartz cuvettes (QS High Precision Cell; 10 mm (Hellma^®^ Analytics, Jena, Germany)). For the characterization of micelles, a Zetasizer Nano ZS from Malvern was used with zeta-potential cells. Sonication was performed using an Elmasonic S 30 H ultrasonic bath (Fisherbrand^®^ FB15051, Waltham, MA, USA) at a frequency of 50/60 Hz. ^1^H NMR spectra were recorded in a Bruker Avance III 300 MHz instrument. Chemical shifts of ^1^H (δ, ppm) are reported relative to the residual solvent peaks relative to SiMe_4_. Infrared spectra were recorded as KBr pellets on a Bruker Tensor 27 spectrometer. HPLC analysis was performed on a Perkin-Elmer Series 200 Pump coupled to a Perkin-Elmer Series 200 UV–Vis Detector. The solvents were of HPLC grade. The column was a SUPELCO Analytical (Discovery^®^, Seattle, WA, USA) BIO Wide Pore 300 Å, C18; 25 cm × 4.6 mm, 5 μm (Sigma-Aldrich^®^, St. Louis, MI, USA). The mobile phase was composed of trifluoroacetic acid (TFA) 0.1% aqueous (A) and acetonitrile with 0.1% TFA (B), at a flow rate of 1 mL min^−1^. For PQ analysis, UV detection was performed at 272 nm and the gradient elution program was 10–70% B from 0 to 15 min, 70–10% B from 15 to 18 min, and 10% B from 18 to 20 min for column re-equilibration. For AuSe and AuS analysis, UV detection was performed at 290 nm and the gradient elution program was 40–100% B from 0 to 15 min and 100% B from 15 to 20 min. The quantification of the encapsulated drugs was achieved with reference to a standard calibration curve (Supplementary Materials, Figure S1).

### 2.3. Synthesis and Characterization of MePEG-b-PCL

The copolymer methoxy-terminated poly(ethylene glycol)-*b*-poly(ε-caprolactone) (MePEG-*b*-PCL) was synthesized following well-established methods described in the literature [20,21,22,23]. Briefly, MePEG-b-PCL was synthesized by metal-free cationic ring-opening polymerization of ε-caprolactone (ε-CL) via an activated monomer mechanism in the presence of HCl-diethyl ether using methoxy-terminated poly(ethylene glycol) (HO-PEG-Me) as macroinitiator. To 5.0 g of MePEG (1.0 mmol, M_n_ = 5000) dried twice by azeotropic distillation in toluene was added dried DCM (50 mL), 5.0 g of distilled ε-caprolactone (43.8 mmol), and 3 mL of 1 M HCl (3 mmol) in diethyl ether and the reaction mixture was maintained overnight under nitrogen and vigorous stirring at room temperature. After 17 h, the reaction mixture was slowly poured into cold (4 °C) diethyl ether (placed in an ice bath) to finish the polymerization and precipitate the copolymer that was recovered by filtration, re-dissolved in DCM, and dried in a vacuum, to obtain a white solid [20,21,22,23]. MePEG-*b*-PCL was obtained in high yield (93%). The molecular weight of the PCL block was calculated based on the ^1^H NMR spectra and taking into consideration the known molecular weight of the MePEG precursor.

MePEG-*b*-PCL ^1^H NMR (CDCl_3_, δ ppm) assignments were as follows: The resonances assigned to the sequence of the methylene protons of the ester carbonyl moiety of PCL were observed at 1.36 ppm (2H, m, CO-CH_2_-CH_2_-CH_2_-CH_2_-CH_2_-O), 1.63 ppm (4H, m, CO-CH_2_-CH_2_-CH_2_-CH_2_-CH_2_-O), 2.28 ppm (2H, t, CO-CH_2_-CH_2_-CH_2_-CH_2_-CH_2_-O), and 4.06 ppm (2H, t, CO-CH_2_-CH_2_-CH_2_-CH_2_-CH_2_-O). The resonances assigned to the terminal methoxy protons and methylene protons of MePEG were observed at 3.35 ppm (3H, s, CH_3_-O-CH_2_-CH_2_) and 3.63 ppm (4H, s, -O-CH_2_- CH_2_-), respectively. Finally, the terminal methylene protons of PCL were observed at 3.78 ppm (2H, t, CH_2_-CH_2_-OH), and the terminal methylene protons for MePEG at 4.20 ppm (2H, O-CH_2_-CH2-OCO).

IR (KBr) (υ/cm^−1^): 3452 cm^−1^ (-OH, stretching vibration, PCL); 2945 cm^−1^ (C-H, stretching vibration, PCL); 2868 cm^−1^ (C-H, stretching vibration, PEG); 1726 cm^−1^ (C=O, stretching vibration, PCL); 1642 cm^−1^ (N-H, bending vibrations, PEG); 1471 cm^−1^, 1420 cm^−1^, 1399 cm^−1^, and 1368 cm^−1^ (C-N and C-O-H, polymer backbone); and 1193 cm^−1^ and 1171 cm^−1^ (O-C-O and C-O, polymer backbone).

### 2.4. Synthesis of Micelles

BCMs, PQ-BCMs, AuSe-BCMs, and AuS-BCMs were synthesized by the thin-film hydration method [24]. MePEG-*b*-PCL (50 mg, 5.0 μmol) and, for the loaded micelles, PQ (2.0 mg), AuSe (2.0 mg), or AuS (2.0 mg), were dissolved in 5 mL of DMF and, after 4 h under continuous stirring at room temperature (RT), the solvent was evaporated under nitrogen in order to form a thin film. Afterward, it was left under vacuum to guarantee that all the DMF was evaporated. Finally, the film was hydrated with 3 mL of MilliQ water at 60 °C, vortexed, and sonicated for 20 min at 60 °C. The mixture was then maintained under stirring for 4 h at RT. Afterward, the solution was centrifuged at 1000× *g* for 10 min and the supernatant was lyophilized (time (t) = 24 h, temperature (T) = −98 °C, final pressure (P) = 0.001 hPa).

### 2.5. Characterization of Micelles

The hydrodynamic diameter (D_h_) and zeta potential of micelles were determined using a Zetasizer Nano ZS from Malvern with zeta-potential cells. Before the measurements, the micelles were dissolved in MilliQ water in order to obtain 1.0 g L^−1^ solutions that were then sonicated. The micelles were subsequently diluted to obtain 0.1 g L^−1^ solutions and filtered through a 0.20 μm SARTORIUS syringe filter. The particle size was measured by dynamic light scattering (DLS) at 25 °C with a 173° scattering angle and an optic arrangement known as non-invasive back scatter (NIBS). All measurements were performed in triplicate.

### 2.6. Drug Loading Content and Efficiency

The loading content (LC) and loading efficiency (LE) of PQ-BCMs, AuSe-BCMs, and AuS-BCMs were estimated by UV–Vis spectrophotometry by measuring the absorbance at 364, 393, or 364 nm, respectively, with reference to a calibration curve established for the corresponding compounds (Figure S1, Supplementary Materials). For this, 2–3 mg of PQ-BCMs, AuSe-BCMs, or AuS-BCMs were dissolved in 1 mL of DMF, vortexed, and centrifuged at 3000× *g* for 10 min to precipitate the copolymer. The supernatant was then collected and analyzed by UV–Vis spectrophotometry.

The LC was calculated as the ratio of the loaded drug entrapped within the micelles over the total amount of micelles and LE was calculated as the ratio of the entrapped drug over the total amount of drug used to prepare the micelles [24].

### 2.7. Stability Studies

The stability of PQ, AuSe, and AuS encapsulated in the micelles was evaluated by HPLC and UV–Vis spectrophotometry immediately after the preparation and lyophilization of the micelles and 16 months after storage of the lyophilized BCMs at RT. For this, 1–3 mg of PQ, AuSe, AuS, PQ-BCMs, AuSe-BCMs, and AuS-BCMs was dissolved in ACN for the HPLC analysis or DMF, for UV–Vis spectrophotometry, vortexed and centrifuged at 3000× *g* for 10 min to precipitate the copolymer. The supernatant was collected and analyzed. The loading content of the lyophilized micelles stored at RT was also evaluated immediately after preparation and lyophilization and also after 16 months, following the procedure described in Section 2.5.

### 2.8. In Vitro Release Studies

The in vitro release of PQ, AuSe, and AuS from PQ-BCMs, AuSe-BCMs, and AuS-BCMs, respectively, was evaluated at pH 7.4 using the dialysis method [25,26]. Briefly, a solution of 3.0 mg of loaded micelles in 3.0 mL of 0.01 M phosphate buffer saline (PBS) pH 7.4 was placed in a Pur-A-Lyzer^TM^ Maxi 25,000 (MWCO = 25 kDa) from Sigma-Aldrich (St. Louis, MI, USA), immersed into 200 mL of 0.01 M PBS pH 7.4 and maintained at 37 °C under continuous stirring. At predetermined time points, 500 μL of the solution inside the dialysis membrane was retrieved, centrifuged at 2348× *g* (5000 rpm), the supernatant collected and lyophilized (t = 24 h, T = −98 °C, p = 0.001 hPa), and the membrane immersed in fresh medium. Afterward, 500 μL of DMF was added to the lyophilized solutions and the resultant solutions were vortexed and centrifuged at 3000× *g* for 10 min to precipitate the copolymer. The supernatant was collected and analyzed by UV–Vis spectrophotometry. The drug release profile was calculated as the percentage of released drug versus time, in which the 100% release corresponds to the total amount of the respective drug entrapped in the micelles.

### 2.9. In Vitro Activity of Au(III) Bis(Dithiolene) Complexes on the Hepatic Stage of P. berghei Infection

The compounds’ in vitro activity against *P. berghei* infection of a human hepatoma (Huh7) cell line was assessed using a bioluminescence assay, as previously described [27]. Briefly, 1 × 10^4^ Huh7 cells per well were seeded in 96 well plates, 24 h prior to infection, in supplemented RPMI medium (10% (*v*/*v*) fetal bovine serum, 1% (*v*/*v*) penicillin/streptomycin, 1% (*v*/*v*) glutamine, 1% (*v*/*v*) nonessential amino acids, and 10 mM 4-(2-hydroxyethyl)-1-piperazineethanesulfonic acid (HEPES), pH 7) and maintained at 37 °C, 5% CO_2_. PQ, AuSe, and AuS stock solutions were prepared at 10 mM in Dimethyl sulfoxide (DMSO), and PQ-BCMs, AuSe-BCMs, and AuS-BCMs stock solutions were prepared at 81, 27.7, and 39.9 μM, respectively, in supplemented RPMI medium containing 50 μg mL^−1^ gentamicin and 0.8 μg mL^−1^ amphotericin B (herein referred to as infection medium). Compounds were serially diluted in infection medium, which was then used to replace the cell-seeding medium, and the plates were incubated for 1 h at 37 °C, 5% CO_2_. *P. berghei* sporozoites genetically modified to express a fusion of the green fluorescent protein (GFP) and luciferase (*P. berghei*_GFP-Luc_) were isolated from the salivary glands of previously infected female *Anopheles stephensi* mosquitoes, added to the cells at a 1:1 proportion, followed by incubation for 48 h at 37 °C, 5% CO_2_. The effect of the different compounds on cell viability was determined using the CellTiter-Blue assay (Promega, Madison, WI, USA), according to the manufacturer’s protocol. The impact of the compounds on *Plasmodium* hepatic infection was assessed by bioluminescence on a Tecan Infinite M200 plate reader, employing the Firefly Luciferase Assay Kit (Biotium, San Francisco, CA, USA) according to the manufacturer’s instructions.

## 3. Results and Discussion

The diblock copolymer methoxy-terminated MePEG-*b*-PCL was synthesized by metal-free cationic ring-opening polymerization of *ε*-caprolactone (CL) via an activated monomer mechanism with HCl-diethyl ether following reported synthetic methodologies described in the literature [23,28]. The copolymer was characterized by ^1^H-NMR and FTIR spectroscopy, being the spectroscopic data collected similar to those described in the literature [23]. The molecular weight (M_n_) of the PCL block was controlled by the feed ratio of CL relative to the MePEG in the reaction mixture. The MePEG-*b*-PCL molecular weight was calculated by ^1^H NMR. The ratio of the integrated peak areas of the resonances attributed to the methylene PEG block (at δ 3.63 ppm; 4H/monomer), and the integrated peak area assigned to the resonance of the PCL block (at δ 2.28 ppm; 2H/monomer) and the molecular weight of the MePEG precursor (M_n_ = 5000 Da; n = 113 monomers) were used to determine the chemical composition and molecular weight of the copolymer. Based on the calculated number of CL monomers (ca. 45), the estimated molecular weight for MePEG-b-PCL was around 9700 Da.

### 3.1. Synthesis and Characterization of Micelles

**Figure 2:**
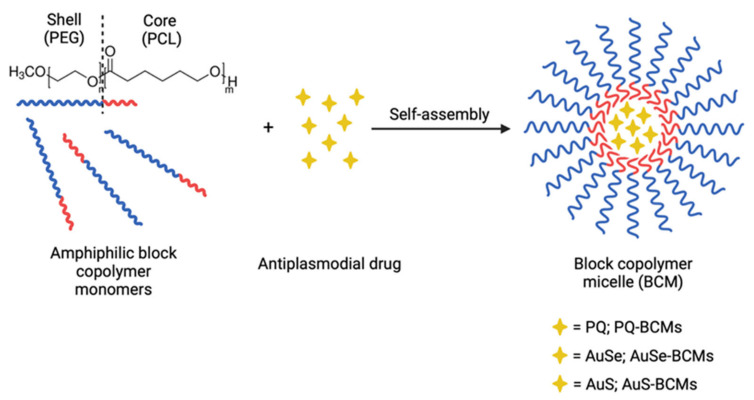
Schematic representation of the synthesis of the micelles with the block copolymer MePEG-*b*-PCL (in which n = 113 and m = 45) and the antiplasmodial drugs.

The gold(III) bisdithiolene complexes AuSe and AuS (Figure 1) differ mainly in the nature of the exocyclic chalcogen atom (selenium vs. sulfur) and both present a similar square-planar geometry. Both complexes exhibited remarkable antiplasmodial activity against the hepatic stage of *Plasmodium berghei* parasites but they are highly lipophilic and may be considered water-insoluble complexes. To overcome their poor water solubility aiming to increase their bioavailability and reduce side effects, we prepared block copolymer micelles (BCMs) loaded with the gold complexes AuSe and AuS. BCMs loaded with the standard antiplasmodial drug PQ were also prepared to be included as a positive control (Figure 1). The non-loaded BCMs and the micelles loaded with the reference compound primaquine (PQ-BCMs) and with the monoanionic Au(III) bis(dithiolene) complexes AuSe (AuSe-BCMs) and AuS (AuS-BCMs), whose structures were previously reported [14], were prepared by the thin-film hydration method [24] (Figure 2).

**Figure 3:**
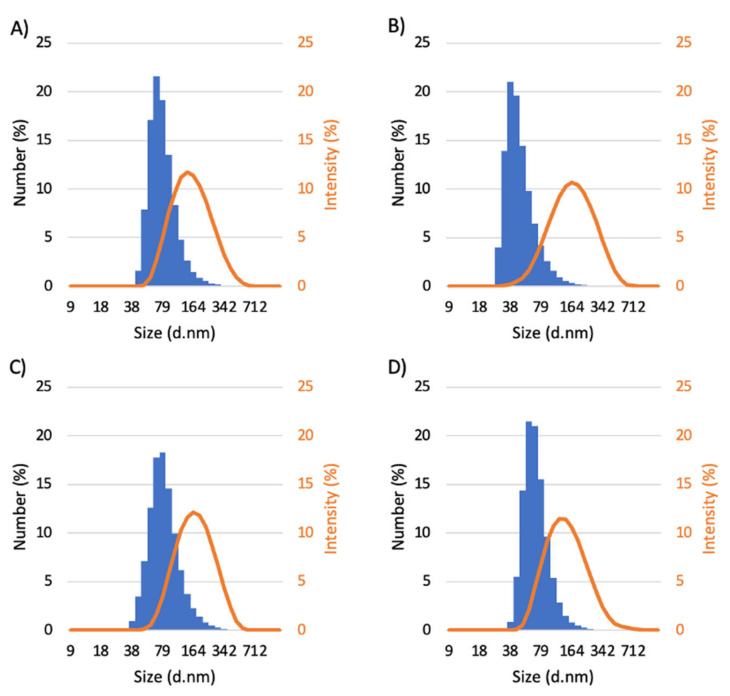
DLS histograms of (**A**) BCMs; (**B**) PQ-BCMs; (**C**) AuSe-BCMs; and (**D**) AuS-BCMs.

All micelles were characterized by dynamic light scattering (DLS) to determine the relative hydrodynamic diameter (Dh), polydispersity index (PDI), and zeta potential (Table 1, Figure 3).

The size of the nanoparticles is a crucial parameter that can significantly influence their pharmacokinetics and in vivo biodistribution. Therefore, a major barrier for all colloidal drug carriers is non-specific uptake by the reticuloendothelial system (RES). The ability to avoid RES uptake is a key parameter for achieving long residence time in the blood stream. Drug delivery systems that are smaller than 200 nm, in general, have low uptake by the RES and may circulate in the blood for prolonged periods.

The DLS histograms (Figure 3) presented by number (%) and also by intensity (%) revealed a monomodal size distribution for all the polymeric micelles and that the D_h_ are all of the same order of magnitude. The mean hydrodynamic diameters obtained are all below 200 nm, which is considered promising for a long circulation half-life, as discussed above [29]. The low polydispersity indexes (PDI), all of them between 0.18 and 0.22, suggest that the formulations were homogeneous and comparable to other micelles synthesized from the copolymer PEG-*b*-PCL [28,30,31,32]. Moreover, the high absolute values found for the zeta potential are indicative of high stability and a low tendency to aggregate.

### 3.2. Drug Loading Content and Efficiency

The loading content (LC) and loading efficiency (LE) were determined by UV–Vis spectrophotometry with reference to standard calibration curves established for the respective drugs (Figure S1, Supplementary Materials) and were calculated as the ratio of the weight of the drug entrapped within the micelles and the total weight of the micelles, and as the ratio of the weight of the drug entrapped within the micelles and the total weight of drug used, respectively [24]. For PQ-BCMs, the drug LC was 3.7% and the LE was 82.5%. For AuS-BCMs, the results were similar, with an LC of 3.8% and a LE of 77.4%. The LC and LE decreased to 2.9% and 55.5% for AuSe-BCMs (Table 1). Micellar cores may be seen as nano-reservoirs for loading hydrophobic compounds that are encapsulated in the core. The ability to incorporate drugs in polymeric micelles is dependent on several factors, including the length of the core and shell-forming blocks in the copolymer, relative concentrations of drug to be incorporated, and the polymer, among others. Anyway, the ability to incorporate high cargoes of drugs in the micelles is always a challenge and may be optimized for each drug. To increase the loading content and efficiency, the preparation of the loaded micelles included a sonication step at 60 °C that was crucial to achieving the presented loading contents.

### 3.3. Stability Studies

**Figure 4:**
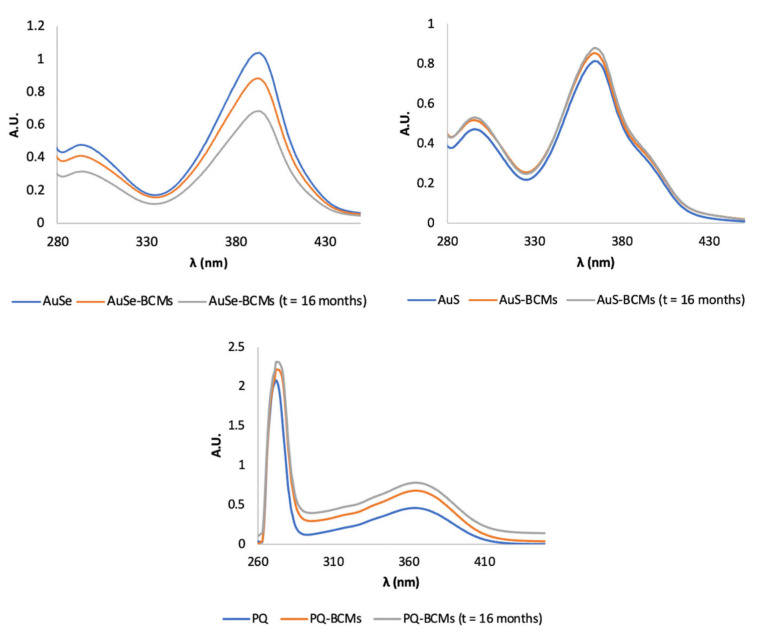
Comparison between the UV–Vis spectra of free AuSe, AuS, and PQ (blue lines) and the respective loaded micelles (AuSe-BCMs, AuS-BCMs, and PQ-BCMs) immediately after the preparation of the micelles (orange lines) and after 16 months (gray lines).

**Figure 5:**
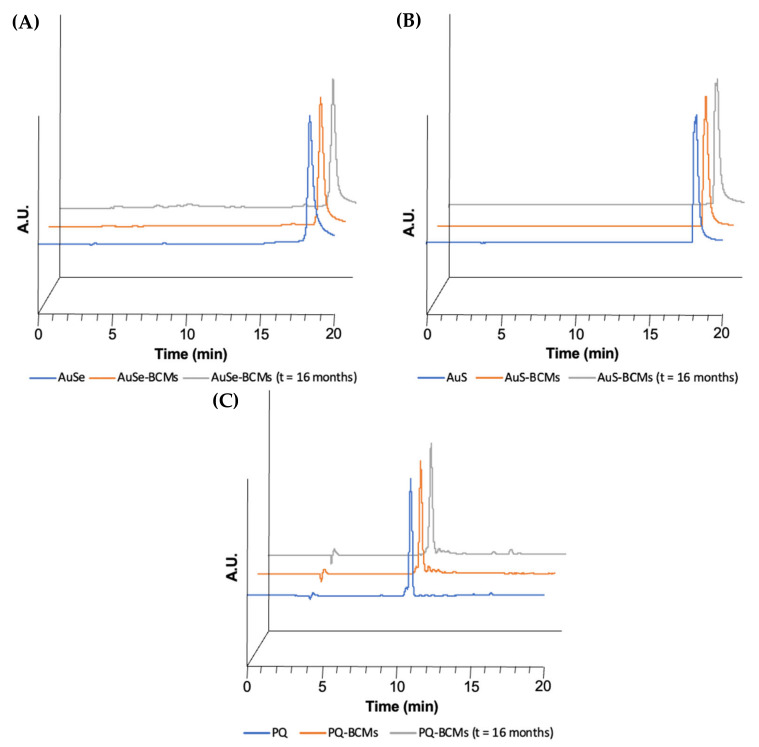
HPLC chromatograms of (**A**) AuSe (R_t_ = 18.35 min), AuSe-BCMs immediately after the preparation (R_t_ = 18.35 min), and AuSe-BCMs after 16 months (R_t_ = 18.45 min) with UV detection at 290 nm; (**B**) AuS (R_t_ = 18.24 min), AuS-BCMs immediately after the preparation (R_t_ = 18.15 min) and AuS-BCMs after 16 months (R_t_ = 18.19 min) with UV detection at 290 nm; and (**C**) PQ (R_t_ = 11.05 min), PQ-BCMs immediately after the preparation (R_t_ = 11.04 min) and PQ-BCMs after 16 months (R_t_ = 10.96 min) with UV detection at 272 nm.

The stability of the drugs encapsulated in the micelles was qualitatively evaluated by UV–Vis and HPLC immediately after the preparation and lyophilization of the micelles and 16 months after the storage of the lyophilized micelles at RT. To this end, the loaded micelles were disassembled with DMF or ACN, centrifuged to precipitate the copolymer, and the supernatant was collected and analyzed. The free PQ, AuSe, and AuS and the drugs collected from the loaded micelles (PQ-BCMs, AuSe-BCMs, and AuS-BCMs) immediately after the synthesis and lyophilization presented identical absorption spectra and chromatographic profiles, with similar retention times and chromatographic profile, suggesting that the drugs maintain their chemical structure unaltered without suffering degradation after being encapsulated in the micelles and exposed to the conditions used to prepare the micelles (Figure 4 and Figure 5). In addition, 16 months after lyophilization, the BCMs stored at RT were analyzed once again, with identical results, demonstrating the stability of the micelles and the incorporated drugs over time.

The loading content of PQ-BCMs, AuSe-BCMs, and AuS-BCMs was also determined at the end of the stability study following the same procedure employed immediately after the preparation and lyophilization (Table 2). The loading content of the micelles remained unaltered, validating the stability of the lyophilized micelles.

### 3.4. In Vitro Release Studies

The stability of the polymeric micellar structure is crucial to control the rate of drug release. The release of drugs physically encapsulated in polymeric micelles may be considered dependent on the rate of drug diffusion in the micellar core or by the disassembly of the micelles [33]. The drug diffusion rate may be relatively low if favorable interactions occur between the entrapped drug and the polymeric chain of the core-forming block. Hydrogen bonds are expected to occur between the BCMs and the entrapped gold complexes AuS and AuSe which may slow the release rate enhancing the kinetic stability. Moreover, the MePEG-b-PCL copolymer has a very low critical micelle concentration (CMC) of 4 mg L^−1^ as previously described by us [28]. Polymeric micelles self-assembled from copolymers with low CMC tend to present higher thermodynamic stability even under highly diluted conditions in the blood stream. Therefore, the rate of drug release is a crucial parameter that should be determined for all the micellar formulations. Dialysis is the most frequent method used to determine the in vitro drug release profile of different types of nanoparticles including polymeric micelles [34].

**Figure 6:**
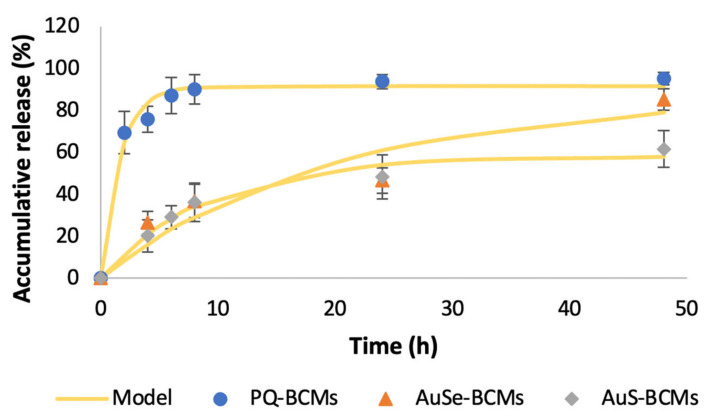
In vitro PQ, AuSe, and AuS release profile from PQ-BCMS, AuSe-BCMs, and AuS-BCMs, respectively, at pH 7.4 and mathematical modeling of the in vitro release profile from the micelles, using a first-order model.

The in vitro PQ, AuSe, and AuS released from PQ-BCMs, AuSe-BCMs, and AuS-BCMs, respectively, were evaluated by dialysis in physiological conditions (pH 7.4, 37 °C). In the first two hours, PQ-BCMs released 69% of the drug, while the micelles loaded with the gold compounds (AuSe and AuS) only released around 30% in the same time period. Nonetheless, it is important to note that a first-order model (Equation (1), Table 3 and Figure 6) [35], where *F*_%_ is the fraction of the accumulated drug released at time *t*, *F_max_* is the maximum amount of drug released and *k* is the first-order release constant, presents a good fit for the data obtained.

(1) $$F_{\%}=F_{max}\left(1-e^{-kt}\right)$$

Despite dialysis assays being the most prevalent method used to evaluate drug release, the validity of the method as well as the reliability of the release data generated from dialysis assays should be carefully analyzed [34]. In fact, the released drug needs to diffuse through the dialysis membrane affecting the measured drug release profile. Since the gold complexes, AuS and AuSe, are highly insoluble in water, evaluating the drug release profile of these compounds is very challenging. We have determined the release profile based on the dialysis method but we have measured the drug that remains entrapped inside the micelles aiming to overcome this problem. Nonetheless, we have experienced some experimental limitations, with some difficulties with quantification being faced. For the quantification using UV–Vis spectrophotometry, our UV–Vis cells require a minimum volume of 500 μL, which forced us to withdraw more than 80% of the total volume of the sample over time to allow proper quantification. This may affect the kinetic release process, so further studies should be performed in other experimental conditions to avoid the potential effect of the high volume reduction. Furthermore, we have observed that after the release of AuSe and AuS complexes from the micelles, they tend to precipitate inside the dialysis membrane due to their very low water solubility. The low aqueous solubility of AuSe and AuS gold complexes (<1 mg/100 mL), together with the fact that the Pur-A-Lyzer^TM^ Maxi 25,000 dialysis membranes only allow a total volume of 3 mL inside the dialysis membrane, did not allow us to carry out the experiments in sink conditions. PQ, on the other hand, has higher water solubility (50 mg/mL; Sigma-Aldrich) and so, in this case, the experiment was carried out in sink conditions. Taking this into consideration, the results should be carefully analyzed and compared, since PQ is not in the same conditions as the AuSe and AuS complexes. However, given the limitations aforementioned, we believe that the experimental procedure used can adequately describe the release of the gold complexes since they are soluble while in the form of micelles and, upon release, they lose their solubility and we efficiently separate the encapsulated compounds from the already released drug by centrifugation.

These release profiles are similar to those described in the literature for other loaded micelles prepared from the MePEG-*b*-PCL copolymer [31,32].

### 3.5. In Vitro Activity of Au(III) Bis(Dithiolene) Complexes on the Hepatic Stage of P. berghei Infection

The antiplasmodial hepatic activity of AuSe and AuS, with and without encapsulation in BCMs, was evaluated in vitro. PQ, the gold standard drug to treat liver-stage *Plasmodium* infection, and the only one known to eliminate *P. vivax* dormant forms—hypnozoites—was used as a positive control [36]. In experiments where the compounds were encapsulated, BCMs alone were employed as negative controls. Human hepatoma cell lines and rodent *Plasmodium parasites* are the most commonly employed system to assess the hepatic stage activity of antiplasmodial compounds, as thoroughly reviewed in [37].

**Figure 7:**
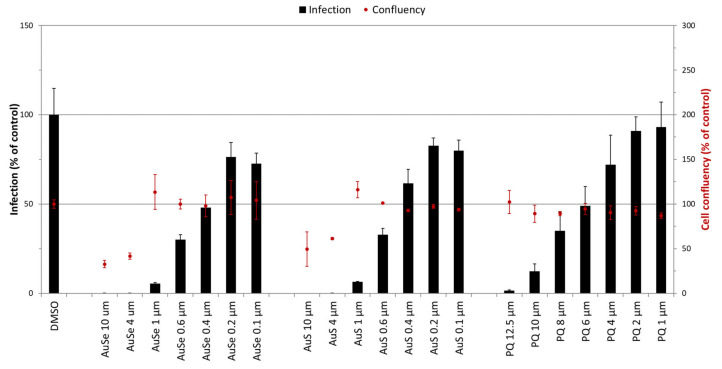
In vitro activity of Au(III) bis(dithiolene) complexes against *P. berghei* hepatic infection. The inhibitory activity of primaquine (PQ), [PPh_4_][AuSEt(=Se)] (AuSe), and [PPh_4_][AuSEt(=S)] (AuS) is represented as a percentage of relative luminescence units of DMSO control. Compound cytotoxicity was measured by fluorescence following AlamarBlue addition and normalized to the DMSO control. Results are expressed as a mean ± the standard deviation.

Our results show that both AuSe and AuS were active against *P. berghei* hepatic infection at all concentrations employed and that their inhibitory activity was higher than that of the PQ control (Figure 7); however, both AuSe and AuS displayed cytotoxic activity at 10 μM and 4 μM, reflecting potential issues that might arise from the administration of therapeutic concentrations of the compounds in humans.

**Figure 8:**
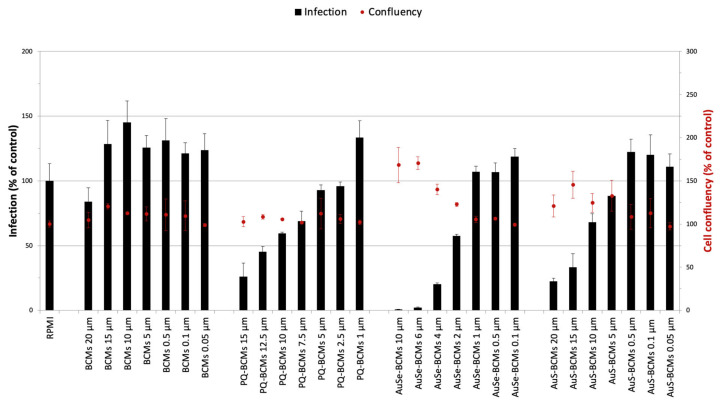
In vitro activity of encapsulated Au(III) bis(dithiolene) complexes against *P. berghei* hepatic infection. The inhibitory activity of primaquine-block copolymer micelles (PQ-BCMs), [PPh_4_][AuSEt(=Se)]-block copolymer micelles (AuSe-BCMs), and [PPh_4_][AuSEt(=S)]- block copolymer micelles (AuS-BCMs) is represented as a percentage of relative luminescence units of BCMs control. Compound cytotoxicity was measured by fluorescence following AlamarBlue addition and normalized to the RPMI control. Results are expressed as a mean ± the standard deviation.

Compounds evaluated in their encapsulated form displayed lower activity against *P. berghei* hepatic infection than their non-encapsulated counterparts (Figure 8). Nevertheless, at the highest screened concentrations of 10 μM for AuSe and 20 μM for AuS, no cytotoxic activity was observed. Of notice, at 20 μM the BCMs employed as negative control displayed some degree of inhibitory activity against hepatic infection.

Compared with the positive control, AuS-BCMs present similar activity, while AuSe-BCMs exhibit a significantly higher antiplasmodial activity, with almost no hepatic infection for activities above 6 μM. These two monoanionic gold bis(dithiolene) complexes exhibit similar geometries with the same counter ions, the only difference relies on the nature of the exocyclic chalcogen atoms. The presence of exocyclic selenium atoms, instead of sulfur ones, increases the efficiency of these complexes as an antiplasmodial drug. In fact, the presence of selenium in metal-based complexes has been shown to confer anticancer and antimicrobial properties [38]. Selenium compounds have also shown promising results in parasitic diseases. The mechanism of activity is not clear yet but it seems that Se can target different parasite’s essential pathways upon interaction with selenoproteins and thiol groups that cause the impairment of antioxidant enzymes and proteins, leading to disruption of antioxidant systems [39,40].

Overall, these results suggest that the encapsulation of these gold complexes in BCMs can improve their release profile and enhance their biocompatibility for administration to humans as antiplasmodial therapeutics.

## 4. Conclusions

Non-loaded BCMs and BCMs loaded with PQ, [PPh_4_][AuSEt(=Se)] (AuSe), and [PPh_4_][AuSEt(=S)] (AuS) were successfully synthesized from the copolymer MePEG-*b*-PCL and fully characterized. These BCMs were obtained with mean hydrodynamic diameters below 200 nm, and monomodal size distribution and zeta potential values in the range −57.3–−38.6 mV, suggesting a low tendency of the nanoparticles to aggregate. The antiplasmodial drugs did not suffer degradation after encapsulation of the micelles and the BCMs maintained their stability for up to 16 months in the lyophilized form stored at room temperature. The loaded-BCMs were obtained with loading efficiency in the range of 55.5–82.5%. The in vitro release studies performed demonstrate a sustained release of our monoanionic gold (III) bis(dithiolene) complexes, an important feature to improve the circulation half-life of the drugs.

In vitro studies of the antiplasmodial hepatic activity of the gold(III) complexes and the positive control PQ, along with their encapsulated counterparts, indicate that all formulations are active against *P. berghei* hepatic infection, with the non-encapsulated forms of the drugs being more active. Compared with the positive control PQ, both AuSe and AuS present a higher inhibitory activity, while in the case of their encapsulated forms, AuSe-BCMs still exhibit a significantly higher antiplasmodial activity.

Taken together, these results point towards the importance of these monoanionic gold(III) bis(dithiolene) complexes as prospective antiplasmodial drugs and the need for continuous research toward the future design and development of other related complexes in an attempt to link drug structures or properties to antiplasmodial activities.

The use of BCMs as drug delivery vehicles for these novel complexes could not only overcome the emergence of drug resistance to conventional antimalarial drugs but also enhance their bioavailability and biocompatibility, thereby improving their pharmacologic and therapeutic properties.

## Figures and Tables

**Table 1:** Hydrodynamic diameter (D_h_), Polydispersity index (PDI), Zeta Potential, Loading Content (LC), and Loading Efficiency (LE) of micelles.

Micelles	D_h_ (nm)	PDI	Zeta Potential (mV)	LC (%)	LE (%)
BCMs	82.1 ± 2.7	0.21 ± 0.02	−41.1 ± 0.2	-	-
PQ-BCMs	50.9 ± 2.8	0.22 ± 0.01	−43.6 ± 1.6	3.7 ± 0.3	82.5 ± 0.7
AuSe-BCMs	87.1 ± 9.7	0.18 ± 0.004	−38.6 ± 0.8	2.9 ± 0.3	55.5 ± 0.7
AuS-BCMs	72.8 ± 3.1	0.18 ± 0.02	−57.3 ± 0.7	3.8 ± 0.4	77.4 ± 0.8

**Table 2:** Loading content of PQ-BCMs, AuSe-BCMs, and AuS-BCMs immediately after the preparation of the micelles (t = 0) and after 16 months (t = 16 months).

Micelles	LC (%) t = 0	LC (%) t = 16 Months
PQ-BCMs	3.7 ± 0.3	3.8 ± 0.6
AuSe-BCMs	2.9 ± 0.3	2.8 ± 0.02
AuS-BCMs	3.8 ± 0.4	3.7 ± 0.1

**Table 3:** Parameters and goodness of fit exhibited by the first-order model used to fit the fraction of the accumulated drug release (*F*_%_) as a function of time.

Parameters	PQ-BCMs	AuSe-BCMs	AuS-BCMs
*k*	0.61	0.05	0.11
*F_max_*	91.49	86.66	58.12
R^2^	0.98	0.89	0.98

## Supplementary Materials

The following supporting information can be downloaded at https://www.mdpi.com/article/10.3390/pharmaceutics15031030/s1, Figure S1: Calibration curves of (A) PQ at λ = 364 nm; (B) AuSe at λ = 393 nm; and (C) AuS at λ = 364 nm.
