# Supplementary material for: Block Copolymer Micelles Encapsulating Au(III) Bis(Dithiolene) Complexes as Promising Nanostructures with Antiplasmodial Activity
Source: Pharmaceutics. 2023 Mar 22;15(3):1030. doi: 10.3390/pharmaceutics15031030 (PMC10058554; doi:10.3390/pharmaceutics15031030)
Supplement: Supplementary file 1 [file pharmaceutics-15-01030-s001.zip › pharmaceutics-2203769-supplementary.pdf]

Block Copolymer Micelles Encapsulating Au(III) Bis(dithiolene) Complexes as Promising Nanostructures with Antiplasmodial Activity

Joana F. Santos [1], Raquel Azevedo [2], Miguel Prudêncio [2], Fernanda Marques [1,3], Yann Le Gal [4], Dominique Lorcy [4], Célia Fernandes [1,3]*

[1] Centro de Ciências e Tecnologias Nucleares, Instituto Superior Técnico, Universidade de Lisboa, Estrada Nacional 10, km 139.7, 2695-066 Bobadela LRS, Portugal

[2] Instituto de Medicina Molecular João Lobo Antunes, Faculdade de Medicina, Universidade de Lisboa, Av. Prof. Egas Moniz, 1649-028 Lisboa, Portugal

[3] Departamento de Engenharia e Ciências Nucleares, Instituto Superior Técnico, Universidade de Lisboa, Estrada Nacional 10, km 139.7, 2695-066 Bobadela LRS, Portugal

[4] Institut des Sciences Chimiques de Rennes-UMR 6226, Université de Rennes, CNRS, ISCR, F-35000 Rennes, France

Table of Contents

Figure S1. **2**

PQ, AuSe and AuS were dissolved in DMF, to afford 2.0 g L^{-1} solutions. These solutions were diluted to prepare serial concentrations corresponding to 0.01 – 0.06 mg mL^{-1}, in the case of PQ, and 0.001 – 0.02 mg mL^{-1}, in the case of AuSe and AuS. The solutions were analyzed by UV-Vis spectrophotometry and the calibration curves were established.

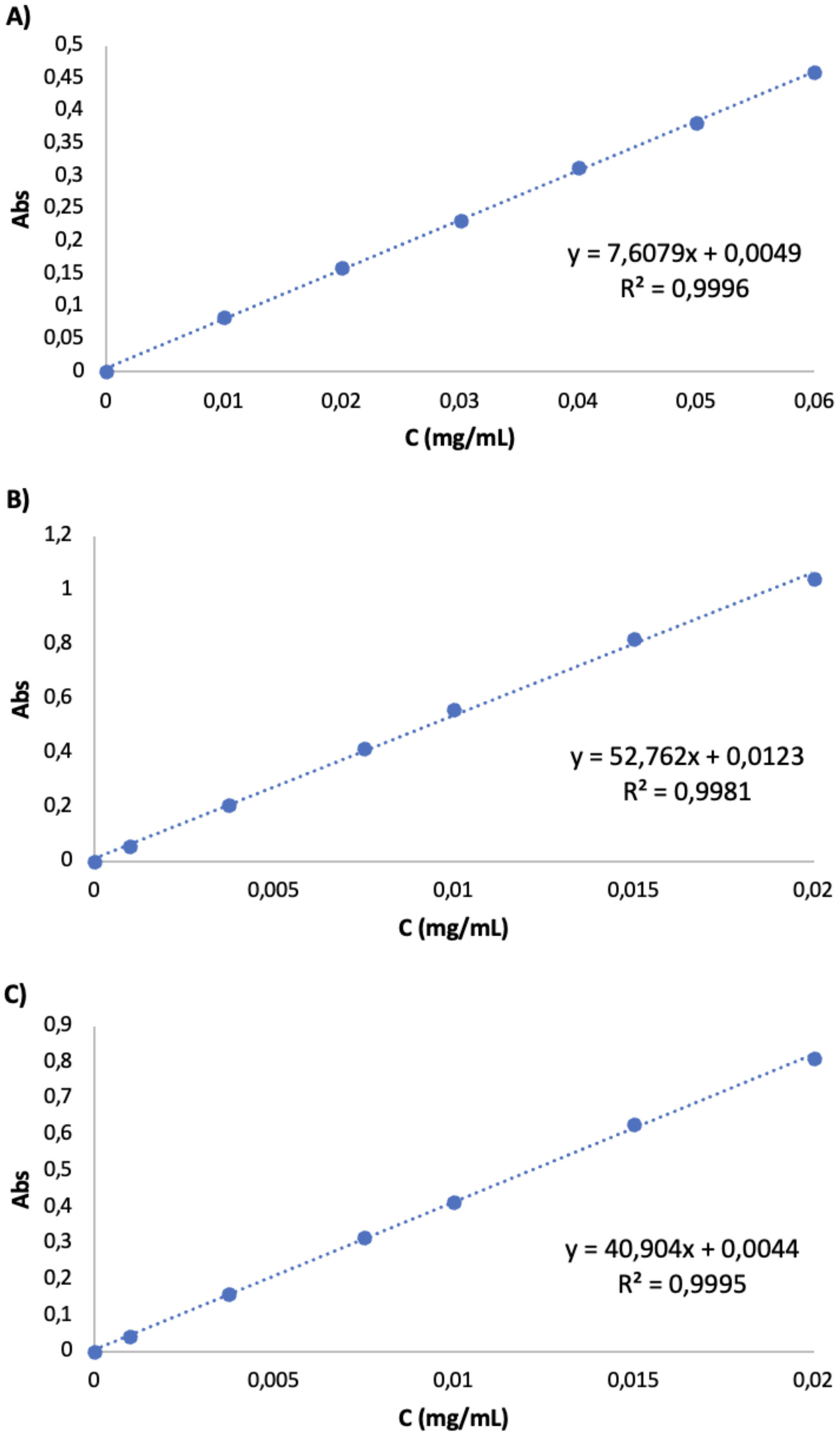

Figure S1. Calibration curves of **A)** PQ at λ = 364 nm; **B)** AuSe at λ = 393 nm and **C)** AuS at λ = 364 nm.
